# Editorial

**Published:** 2010-08-17

**Authors:** Usha Mohan Das

**Affiliations:** Editor-in-Chief

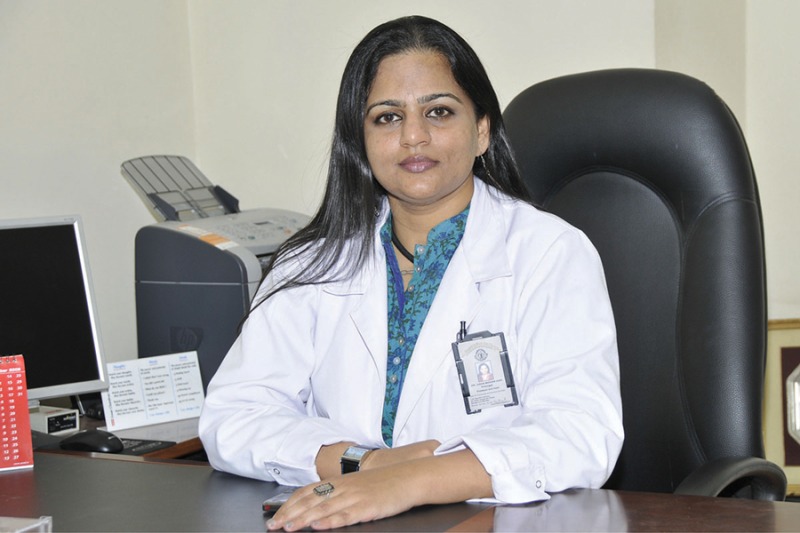



**RESEARCH PARADIGMS.....Near yet so far!!**

Today, the key to our profession is how well we perform when in clinical practice. The true test of a profession’s credibility and growth is the ability of a clinician to get consistent results (what happens and why) in the treatment of patients.

How do we as clinicians, achieve consistent, predictable and reliable results in treatment after treatment, patient after patient? The only answer is “Research”.

All professions grow, change, build credibility and improve based on the research they conduct and produce. Millions of treatment modalities have been delivered. Patients have benefited and moved to new levels of health and wellness.

There exists a voluminous mass of empirical data (mostly from Western statistics) which has produced remarkable results in a large percentage of patients. The question is― Can these results be reproduced reliably and consistently in our country in the existing environment?

Pediatric dentistry is in a stage of growth and development in which a research paradigm must be established and properly conducted. Credible studies must be undertaken, and their results must be verified to validate the results

A number of questions come to mind when asking about research:

- What type of research is going to be conducted?
- Where will it be conducted?
- How will the results of the research impact oral health care delivery?
- Who’s going to fund it?

The gold standard for determining the effectiveness of a product or technique remains the randomized controlled trial (RCT).

While the validity of the RCT has come under fire recently, it is still one of the biggest factors that insurance providers, health plans and managed care groups use to determine whether they would provide coverage. Many of today’s studies are conducted at universities and other institutions. With the advent of the Internet and other forms of communication, there is no reason why our colleagues all over the world and we cannot work together to produce a large, multisite study.

Research helps build the respect and credibility our profession needs. Positive research backed by large, controlled studies can also play a role in the profession’s getting legislations passed that would be favorable to both practitioners and their patients.

Research findings also help protect the public; they can begin to understand what Dentistry is, how it works, and what it can do for them.

If everyone benefits from research, then how come it is not yet happening to a good potential? The most obvious handicap is financial, but there are other reasons too.

Still, much more work needs to be done.

It will not only support and explain procedures; it will lead to better treatment and functional outcomes. For those “sitting on the fence,” it will give them reason to give treatment procedures a try. For those who already believe in oral health care, it will help persuade them to refer more of their friends for care. And for providers of oral health care, research will prove to the world the scientific merit, and help them experience the rewards they so rightly deserve.

**Usha Mohan Das**

Editor-in-Chief

e-mail: ushymohandas@gmail.com

